# News and Miscellany

**Published:** 1905-01

**Authors:** 


### News and Miscellany.

The State Board of Medical Examiners will meet at Austin, May 2, 1905.

Dr. J. S. Brown, Taylor, Texas, graduate of Kentucky School of Medicine, 1883, died at his home December 29th ult.

The "Red Back" sends greeting to its grand army of friends and supporters, and wishes them all a happy and prosperous New Year.

Dr. R. F. Miller, of Sherman, the handsome Treasurer of the State Medical Association is off for New York to take a month's course at the polyclinic.

Drs. F. D. Thompson and Ira C. Chase (Fort Worth)—the handsome Secretary of the State Medical Association—have formed a copartnership. Success to them!

Dr. C. F. Norton, a graduate of the Medical Department of the University of Texas and at one time quarantine officer at El Paso, died recently at Asheville, N. C., of consumption.

Dr. P. C. Coleman, Colorado City, Texas, and Dr. J. O. McReynolds, Dallas, delegates from Texas, represented the State Medical Association at the Pan-American Medical Congress held at Panama January 2-6 inst.

New subscribers are coming in by the dozens. Those who do not catch on now will get left. The "Red Back" will be indispensable this year. It is a warm number; red hot, in fact. Send along your names and your little one dollars.

Dr. Horace Gilbert, surgeon to the Confederate Home at Austin, perpetrated matrimony last month. He was married December 20th at Taylor to the beautiful and accomplished Miss Mabel Pumphry, the acknowledged belle of Williamson county.

Dr. Edmond Souchon, President Louisiana State Board of Health, has been appointed a member of the Executive Council of the American Congress on Tuberculosis, 1905-06. Dr. Denslow Lewis, of Chicago, has been appointed a member of the council.

Dr. C. E. Cantrell, Vice-President State Medical Association of Texas and member of its Committee on Public Policy and Legislation, has been appointed by the Executive Council of the American Congress on Tuberculosis, Vice-President of the Congress for Texas.

"Here's to you and the 'Red Back' another year (20th); the best journal in the South.—J. M. Townes, Joshua, Texas."

"I'm lonesome without the 'Red Back'; change address to El Paso."—F. P. Miller, late of Midland.

I have a complete set of Buck's Reference Hand-Book of Medical Sciences, edition of 1885 (next to the last issued), eight volumes quarto, in sheep, that I will sell for $20. It is second hand, but in good order. Cost, publisher's price, $8.00 per volume, $64. It is a complete library within itself. Some young doctor should have it for the foundation of his library. Speak quick.

DR. W. A. McGEE, Rice, Texas, has been appointed Chairman of the Memorial Committee, State Medical Association, vice Dr. J. E. Thompson, who has declined to serve because of comparatively short residence in Texas, and consequent lack of extended acquaintance amongst the older members, and knowledge of the deceased members.

NEW ORLEANS POLYCLINIC.—Eighteenth annual session opens November 7, 1904, and closes May 10, 1905. Physicians will find the Polyclinic an excellent means for posting themselves upon modern progress in all branches of medicine and surgery. The specialties are fully taught, including laboratory and cadaveric work. For further information, address New Orleans Polyclinic, postoffice box 797, New Orleans, La.

DR. GEO. R. TABOR, State Health Officer of Texas, upon invitation by the faculty, on January 6th inst., delivered before the classes of the University Medical Department at Galveston a lecture upon "The Yellow Fever Epidemic of 1903; the Lessons to be Learned from It." The lecture appeared in full in the *Galveston News* of January 8th. Other lectures will be delivered by distinguished physicians during this year's session upon sanitation, yellow fever, quarantine, etc.

THE SAN ANGELO people have a sensible Business Men's Club. At the November meeting their committee on sanitation, Drs. T. W. Conerly, E. L. Batts, Boyd Cornick and B. F. Lee, made a report on the means of preventing diseases, which is sensible and deals with the subject in a manner easily comprehended by the people. It brings the matter home to every man, and we may expect some municipal acitivity in the way of sanitation. All Texas towns should take an interest in such matters. Many of them are abominally filthy.

MRS. DANIEL begs to acknowledge, with thanks, the receipt, Christmas, of a solid silver souvenir spoon, engraved "Our Mascot," sent anonymously. It came by mail from Lenz Bros., Dallas. She greatly prizes it, and appreciates the thoughtful kindness of her unknown friend, but with a woman's curiosity she would like for him to reveal his identity, that she might thank him personally, and again at Houston next April. Of course, it was sent by one of the doctors of the State Association, amongst whom she is proud to know she has many friends and well wishers.

THE TEXAS EPILEPTIC COLONY, Abilene, Texas, opened for patients in 1904. Report of Superintendent John Preston shows, admitted: Males, 139; females, 85; total, 224; discharged improved, 6; unimproved, 4; furloughed, 7; died, 6. Remaining, August 31, 1904, 201. Of the number admitted 104 were from the three lunatic asylums of the State. The institution is full, and 296 applicants for admittance were refused. Dr. Preston urges the speedy erection of six more cottages to accommodate forty each, and cost $125,000, and other necessary buildings. He advises a brick yard to make the brick for future buildings, such as are in operation in the New York and Alabama colonies, to cost $2500; a $5000 ice plant and a $500 tailor shop. The per capita cost of maintenance was $139.92. The total expense of the colony, $97,979.78. The colony produced $5025.40 worth of stuff—farm products.

THE STATE HEALTH OFFICER's report for the two years ending August 31, 1904, is out. It is most interesting reading, and is very full and complete. I have room this issue for only a brief mention, but will take it up later, "when the hurly burly's done, when the battle's lost and won." Dr. Tabor pays a beautiful tribute to the late Surgeon Murray, Marine Hospital Service. The total appropriation for the department for two years was $108,290. Total expense to the State, two years, $51,090.37. Reverted to treasury, $57,199.63. Earnings of quarantine stations for disinfecting vessels, $38,204.02.

THE TRANSACTIONS of the State Medical Association of Texas has just been issued from the press of the Von Boeckmann-Jones Co., Austin. It is a beautiful volume of 650 pages, and is a model of the bookmaker's art. Moreover, it is a very valuable volume. It contains not only a full report of the proceedings of the big Austin meeting last April—the banner meeting of the Association—but the cream of the papers read and the discussions. It is a volume of high class medical literature, and doubtless will be highly prized by the members. It "lays over" any society transactions that comes to this office. The publishers secured the contract—2550 volumes, 650 pages—on competitive bid, and filled their contract with credit to their great reputation.

